# The analgesic activities of *Stauntonia brachyanthera* and YM_11_ through regulating inflammatory mediators and directly controlling the sodium channel prompt

**DOI:** 10.1038/s41598-017-07095-x

**Published:** 2017-08-08

**Authors:** Dali Meng, Lei Wang, Jingnan Du, Jianchao Chen, Chao Chen, Wei Xu, Chunli Li

**Affiliations:** 1 Key Laboratory of Structure-Based Drug Design and Discovery (Shenyang Pharmaceutical University), Ministry of Education; School of Traditional Chinese Materia Medica, Shenyang Pharmaceutical University, Wenhua Road 103, Shenyang, 110016 PR China; 2 School of Life Science and Biopharmaceutics, Shenyang Pharmaceutical University, Wenhua Road 103, Shenyang, 110016 PR China; 3 Shanghai Star Pharm Co., Ltd, Shanghai, 201318 PR China

## Abstract

The analgesic studies on *Stauntonia brachyanthera*, a traditional Chinese folk medicine used to treat headache, pains and inflammatory diseases in local areas, showed that the EtOH extracts (EESB) and the characteristic ingredient YM_11_ could significantly inhibit the acetic acid-induced writhing responses by 43.1% and 78.95%, and decrease the xylene-induced ear edemas by 48.9% and 21.4%, respectively. EESB could significantly increase pain threshold of mice in hot-plate test, but the effect of YM_11_ was not obviously. Further study in formalin test showed the inhibitory effect of YM_11_ in 2^nd^ phase was more significant than that in 1^st^ phase, revealed the peripheral analgesic activity of YM_11_. The ELISA and Western Blot analysis suggested that the analgesic mechanisms of YM_11_ were related to the inhibitions of the expressions of TNF-α, IL-1β and IL-6, and down-regulations of Na_v_1.8 protein in the left side of L4–6 DRG regulated by MAPKs, in which the levels of p-ERK, p-JNK and p-p38 were all decreased. In addition, the electrophysiological experiments indicated YM_11_ could reduce the Nav1.8 currents by 46.01% in small-diameter DRG neurons. Therefore, the analgesic activity of *S. brachyanthera* might be based on the regulation of inflammatory mediators and the directly control of the sodium channel prompt.

## Introduction

In 2001, International Association for the Study of Pain defined pain as an unpleasant sensory and emotional experience associated with actual or potential tissue damage, or described in terms of such damage. Modern researches show that there are two types of pain: nociceptive and pathological pain. Nociceptive pain is also named as acute pain, which usually accompanies noxious stimuli warning of impending tissue damage. Pathological pain, according to its cause, is divided into inflammatory pain and neuropathic pain, both of which belong to chronic pain. Unfortunately, these types of pain often last quite a long time, even after the precipitating insults have been resolved^1^.

Therefore, the management of pathological pain is a major clinical challenge. Opioids, such as morphine, one of the most commonly prescribed analgesics^2^, is always accompany with many negative side effects, including drug addiction, withdrawal symptom, and respiratory depression, etc. The non-steroid anti-inflammatory drugs (NSAIDs), another commonly used analgesic agent, are also reported for their adverse effect on inhibiting osteoblast growth^3^ and so on. Consequently, continuous efforts in searching for new therapeutic agents with higher safety are inevitably urgent. As is known to all, the traditional Chinese medicines (TCM) have been uses for a long history and have significant therapeutic effects for many diseases due to their multi-constituents, multi-factored and multi-targeted properties, which have obvious advantages for the treatment of diseases with few side effects^4^. The components from those natural resources also have strong biological activities and many of them have been developed into drugs for clinical uses, such as artemisinin, paclitaxel, vinblastinecamptothecine, ginkgolide B, and so on. Therefore, natural products in drug discovery have become a major strategy in modern pharmaceutical research and development, and roughly half of the currently used drugs are directly or indirectly derived from natural products.

*Stauntonia brachyanthera* Hand-Mazz (Lardizabalaceae), an evergreen liana distributing in the southwest of China, has been widely used in the southern provinces of China, especially in the areas of Dong and Yao nationalities, and has been recorded in some medicinal books such as Collection of Chinese Herbal Medicine^5^. Its stems and roots are commonly used as a Dong medicine to treat fever, alleviate dysmenorrhea, relieve pains and handle inflammatory diseases, and the leaves are applied to cure diarrhea, fever and headache^5, 6^. Its fruit, which are known as “zhuyaozi”, are traditionally used to relieve the sufferings of concretion^5^. From these folk medicinal applications, it could be concluded that *S. brachyanthera* has great potential biological activities for the treatments of pain or inflammation.

Up to now, more than 50 nor-oleanane triterpenoids have been isolated from this plant in our previous studies^7, 8^, among which YM_11_ was the most characteristic component because of its highest content. Therefore, with the aim to support its traditional analgesic applications in China, and take full advantage of this valuable medicinal herb, a series of experiments were designed to evaluate the analgesic properties of both *S. brachyanthera* and YM_11_, and reveal their possible mechanisms. Herein, the details of these work will be discussed comprehensively.

## Results

### Compounds elucidations

**Figure 1:**
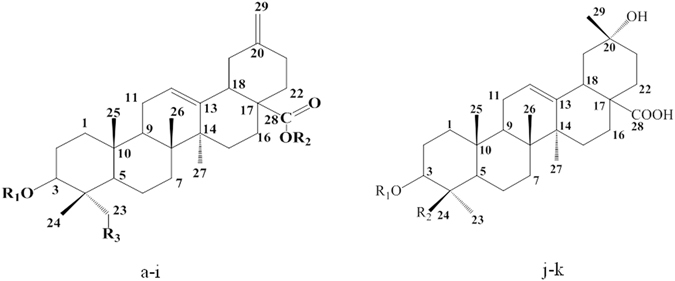
The structures of compounds isolated from the EtOH extracts of *S. brachyanthera*. (a) brachyantheraoside A1 R_1_ = ara- R_2_ = glc1-6glc- R_3_ = OH. (b) brachyantheraoside A3 R_1_ = glcaOMe^1^-^3^glc- R_2_ = rha^1^-^4^glc^1^-^6^glc- R_3_ = OH. (c) brachyantheraoside A4 R_1_ = ara^1^-^4^ara^1^-^3^ara- R_2_ = rha^1^-^4^glc^1^-^6^glc- R_3_ = H. (d) brachyantheraoside A5 R_1_ = ara^1^-(xyl^1^-^2^) ^3^ara^1^-^3^ara- R_2_ = rha^1^-^4^glc^1^-^6^glc- R_3_ = H. (e) YM7 R_1_ = glc^1^-^3^rha^1^-^2^ara- R_2_ = rha^1^-^4^glc^1^-^6^glc- R_3_ = H. (f) YM9 R_1_ = rha^1^-^2^ara- R_2_ = rha^1^-^4^glc^1^-^6^glc- R_3_ = H. (g) YM10 R_1_ = ara- R_2_ = glc^1^-^6^glc- R_3_ = H. (h) YM11 R_1_ = rha^1^-^2^ara- R_2_ = glc^1^-^6^glc- R_3_ = H. (i) YM13 R_1_ = ara- R_2_ = rha^1^-^4^glc^1^-^6^glc- R_3_ = H. (j) brachyantheraoside B6 R_1_ = H R_2_ = rha^1^-^2^glc-O-. (k) brachyantheraoside B9 R_1_ = ara^1^-(rha^1^-^2^) ^3^glc- R_2_ = H.

The detail studies on EESB by various chromatographic methods finally lead to the isolation of 11 compounds. By comparing their ^1^H and ^13^C NMR data with reported values, the structures of these compounds were identify as brachyantheraoside A_1_ (**1**), brachyantheraoside A_3_ (**2**), brachyantheraoside A_4_ (**3**), brachyantheraoside A_5_ (**4**), brachyantheraoside B_6_ (**5**), brachyantheraoside B_9_ (**6**)^8^, YM_7_ (**7**), YM_9_ (**8**), YM_10_ (**9**), YM_11_ (**10**), and YM_13_ (**11**). Their structures were listed in Fig. 1.

### Acetic acid-induced writhing response in mice

**Figure 2:**
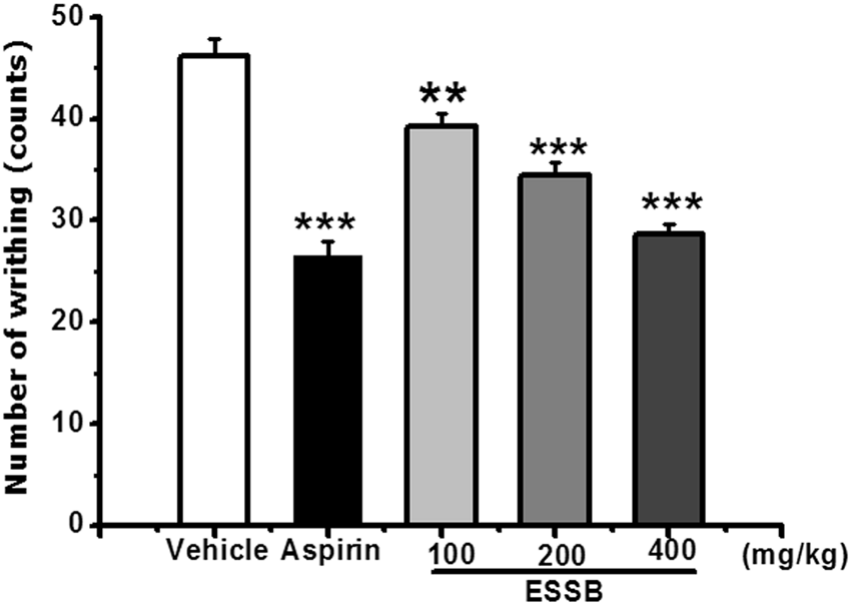
Analgesic effect of EESB on acetic acid-induced writhing response in mice. Results are expressed as mean ± SEM (n = 10). *P < 0.05, ***P < 0.001 *vs*. vehicle.

The statistical data obtained in the study showed that EESB could significantly inhibited the acetic acid-induced writhing response in mice in a dose dependent manner with the inhibition rates of 15.2% (*P* < 0.05), 25.3% (*P* < 0.001) and 43.1% (*P* < 0.001), respectively (Fig. 2).

**Figure 3:**
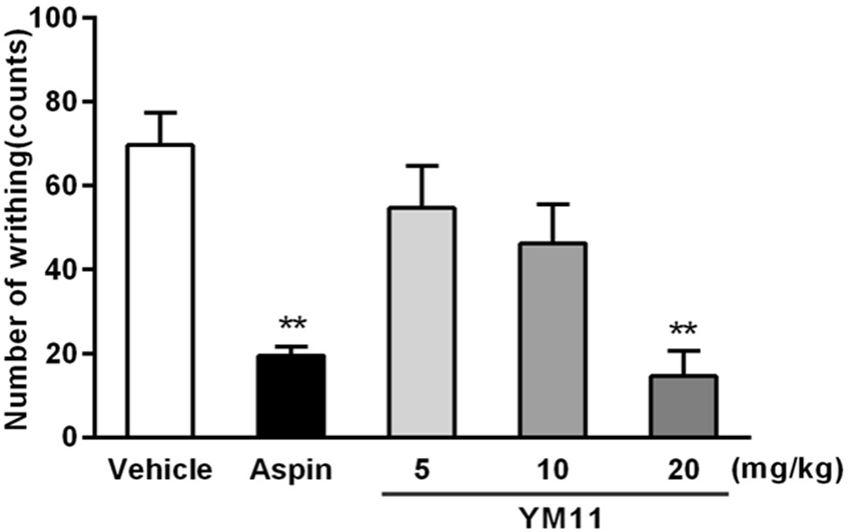
Analgesic effect of YM_11_ on acetic acid-induced writhing response in mice. Results were expressed as mean ± SEM (n = 10). **P < 0.01 *vs*. vehicle.

YM_11_ could also significantly inhibited the writhing response in a dose-dependent manner with the inhibition rates of 21.50%, 33.62% and 78.95% (*P* < 0.01), respectively (Fig. 3). Especially at the dose of 20 mg/kg, the inhibitory rate was higher than that of aspirin (71.87%), suggesting its strong analgesic effect.

### Hot-plate latent pain response test in mice

**Figure 4:**
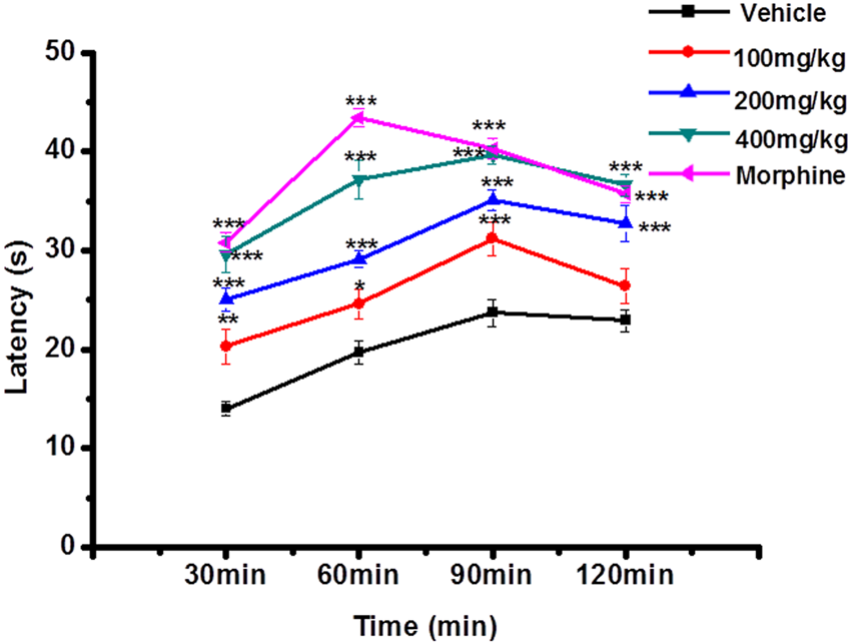
Analgesic effect of EESB as assessed by hot plate test in mice. Results are expressed as mean ± SEM (n = 10). *P < 0.05, **P < 0.01, ***P < 0.001 *vs*. vehicle.

The results of the hot-plate latent pain response test in mice were listed in Fig. 4, which indicated that EESB could significant increase pain threshold of mice in a dose-dependent manner. At the dose of 400 mg/kg, it began to produce antinociceptive effect 30 minutes later (29.6%, *P* < 0.01) of drug administration and reached maximum at 1.5 h (39.6%, *P* < 0.001).

**Figure 5:**
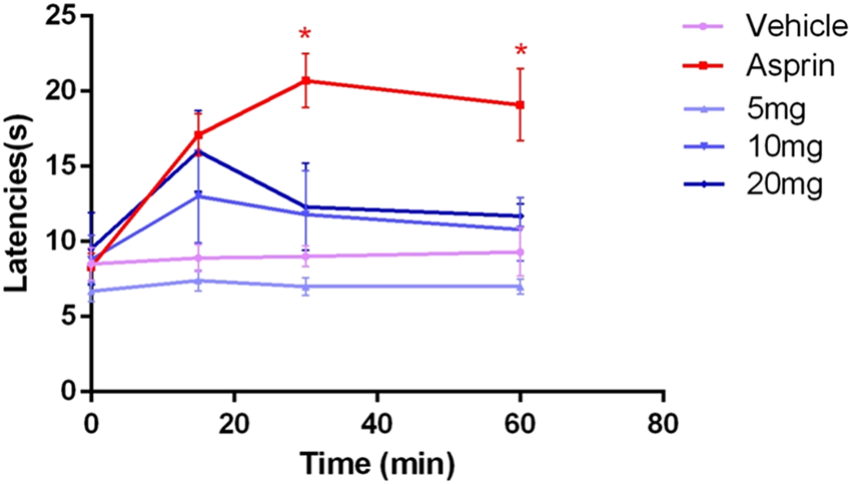
Analgesic effects of YM_11_ (5 mg/kg, 10 mg/kg, 20 mg/kg) at 15, 30 and 60 min after injection, as assessed by hot plate test. Results are expressed as mean ± SEM (n = 10). *P < 0.05 *vs*. vehicle and experimental groups at each time.

While the inhibition rates of YM_11_ at each dose (5 mg/kg, 10 mg/kg, and 20 mg/kg) in this model were 9.7%, 56.7% and 56.7% at 15^th^ min, 5.4%, 40.9% and 40.9% at 30^th^ min, and 5.4%, 26.2% and 26.2% at 60^th^ min, respectively. But there were no significant differences (Fig. 5).

### Xylene-induced ear edema in mice

**Figure 6:**
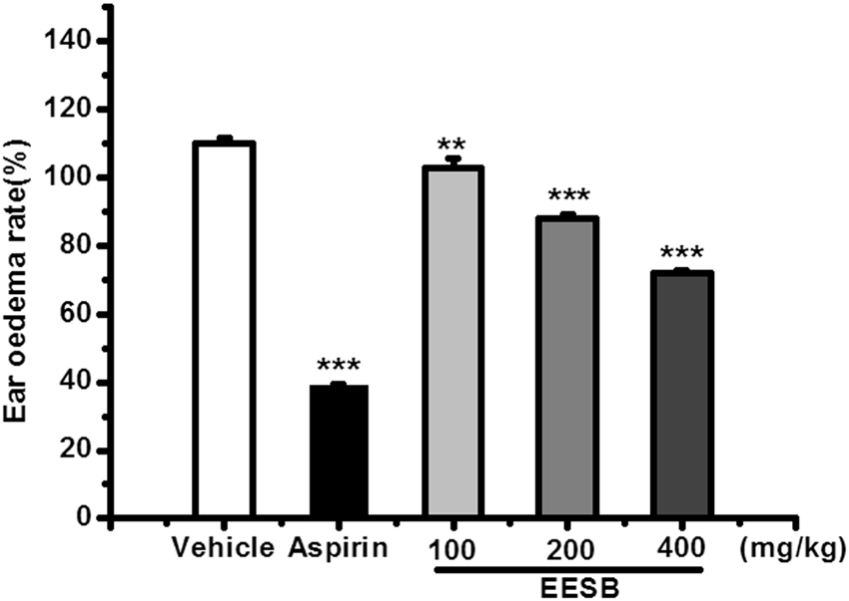
Effect of EESB on xylene-induced ear edema in mice. Results are expressed as mean ± SEM (n = 10). ***P < 0.001 *vs*. vehicle.

**Figure 7:**
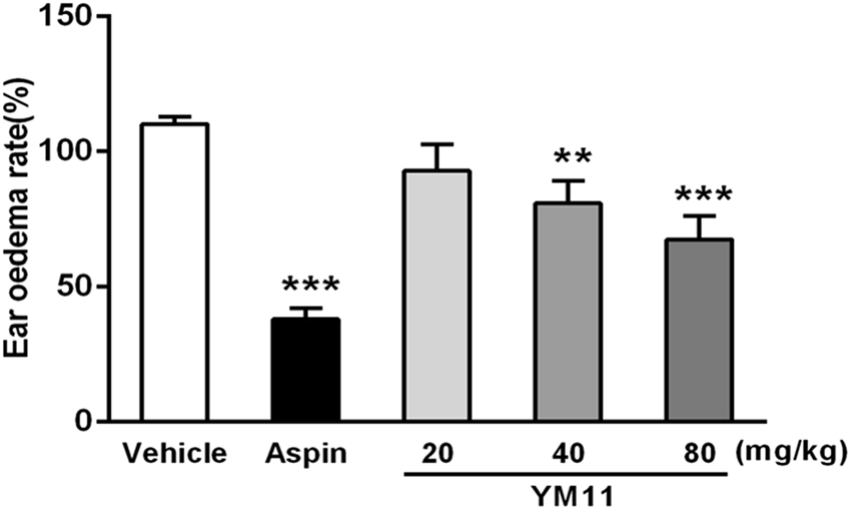
Effect of YM_11_ on xylene-induced ear edema in mice. In each experiment, the both ears of animals were weighted by electronic scales. Values were expressed as mean ± SEM; **P < 0.01, ***P < 0.001 *vs*. vehicle.

EESB could significantly decrease the degree of mouse ear edema compared with control group with a dose-dependent effects (11.9%, 31.8%, and 48.9% at the doses of 100, 200, and 400 mg/kg, respectively. *P* < 0.001), which suggested that EESB may inhibit ear edema by reducing the level of inflammatory mediators during the acute inflammation (Fig. 6). As the characteristic component of EESB, YM_11_ also significantly decreased the ear edemas at the doses of 40 and 80 mg/kg (18.3%, *P* < 0.01, 21.4%, *P* < 0.001) (Fig. 7).

### Formalin test

**Figure 8:**
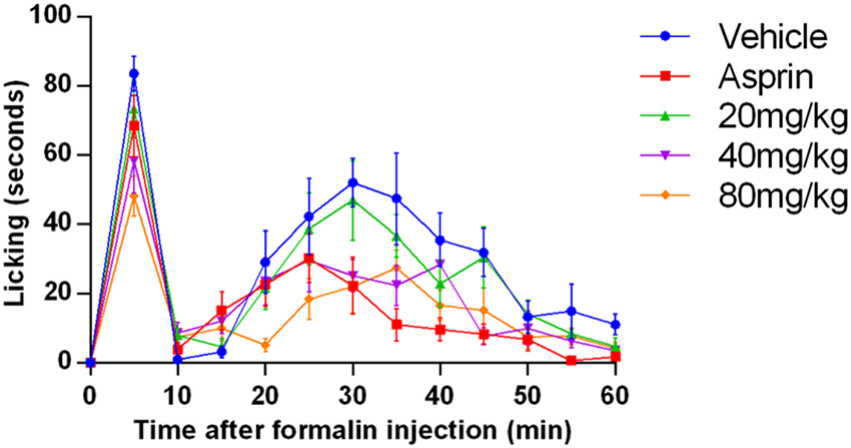
Time course of YM_11_ on formalin-induced inflammatory nociception. The drug or vehicle was intravenous injected 30 min before the formalin injection. Values are the means ± SEM.

**Figure 9:**
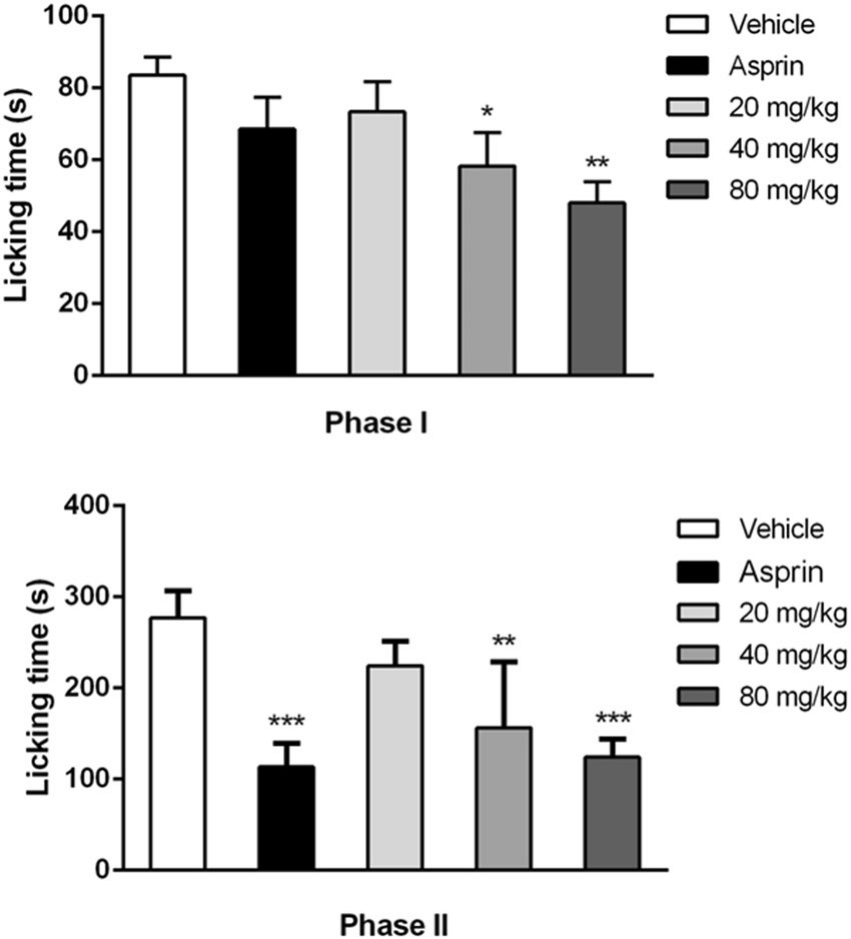
Evaluation of the antinocifensive effect of YM_11_ on phase I (0–5 min) or phase II (15–60 min). Pre-treatment of YM_11_ decreased the time of licking/biting paw. *P < 0.05, **P < 0.01, ***P < 0.001 *vs*. vehicle (n = 12 mice in each group).

The actual results obtained from the test showed that the intra-plantar formalin injection in the control group produced nociceptive pain response, associated with licking (Fig. 8), shaking and retracting, while the local inflammatory reaction, dyskinesia and emotional response were also observed. While these spontaneous pain behaviors and emotional responses were all relieved after the administration of aspirin and YM_11_. As shown in Fig. 9, YM_11_ only showed moderate inhibitory effects on phase I (0–5 min), which were 12.2%, 30.24% (40 mg/kg, *P* < 0.05) and 42.48% (80 mg/kg, *P* < 0.01), respectively. While the inhibitory effects on phase II (15–60 min) were significant compared with control, which were 43.58% at 40 mg/kg (*P* < 0.01) and 55.22% at 80 mg/kg (*P* < 0.001), respectively.

### Biochemical parametersm

**Figure 10:**
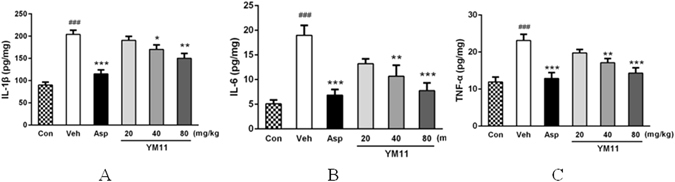
Interleukin-1β (**A**), Interleukin-6 (**B**) and TNF-α (**C**) levels in supernatants of homogenates from xylene-treated ears after treatment with aspirin (200 mg/kg) or YM_11_ (20–80 mg/kg). Measurements were performed with a commercial ELISA kit. Each bar represents the mean ± SEM. The graphic symbols denote the significance levels when compared with control groups. ^###^ *P* < 0.001 *vs*. blank control, *P < 0.05, **P < 0.01, ***P < 0.001 *vs*. vehicle.

The results of the studies on the expression levels of inflammatory mediators in tissue were shown in Fig. 10. Compared with the blank control, the levels of IL-1β (204.12 ± 9.07 pg/mg, *P* < 0.001), IL-6 (16.72 ± 1.41 pg/mg, *P* < 0.001) and TNF-α (23.14 ± 1.67 pg/mg, *P* < 0.001) in the vehicle group were significantly higher, suggesting the successful establishment of experimental models. As the positive control, aspirin inhibit the productions of these inflammatory factors significantly (*P* < 0.001). Compared with vehicle, YM_11_ could effectively inhibit the productions of inflammatory factors in xylene-induced ear edema of mice in dose-dependent manner (170.35 ± 10.26, 10.67 ± 2.23, 17.12 ± 1.13 pg/mg at 40 mg/kg, *P* < 0.05, and 150.23 ± 11.21, 7.77 ± 1.57, 14.32 ± 1.43 pg/mg at 80 mg/kg, *P* < 0.01 for IL-1β and IL-6, *P* < 0.01 for TNF-α, respectively).

## Western Blot Analysis

### Na_v_1.8

**Figure 11:**
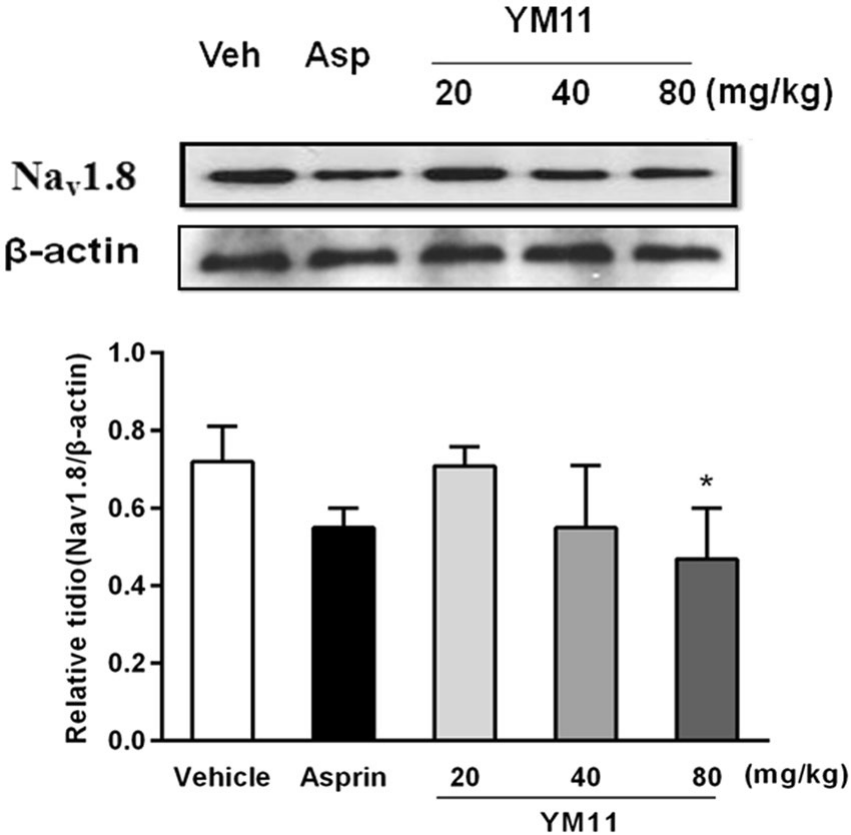
Changes of Na_v_1.8 protein expression in L_4–6_ DRGs among groups. (**A**) The representative bands for the expressions of Na_v_1.8 in DRG after injection of formalin. (**B**) The quantitative data for the expression of Na_v_1.8. The fold change for the density of Na_v_1.8 and normalized to β-actin for each sample. Data were shown as mean ± SEM. *P < 0.05 *vs*. vehicle.

The expression of protein also may be a useful tool to examine the effectiveness of different analgesic regimens^9, 10^. As shown in Fig. 11, compared with solvent control, the expressions of Na_v_1.8 in L4–6 DRGs at aspirin and YM_11_ groups were all decreased with a dose-dependent manner.

### p-MAPKs

**Figure 12:**
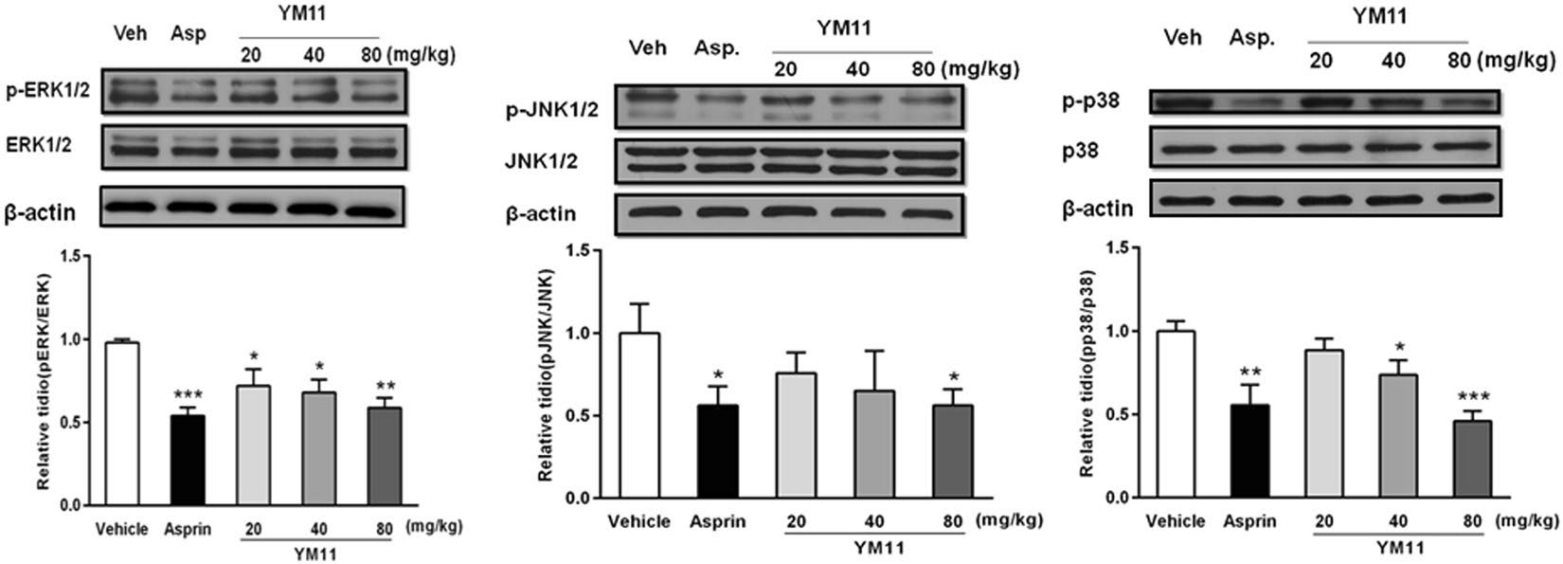
Changes of MAPKs protein expression in L_4–6_ DRGs among groups. The representative bands for the expressions of p-ERK, p-JNK and p-p38 in DRG after injection of formalin. The quantitative data for the expression of each phospho-MAPKs. The fold change for the density of each phospho-MAPKs normalized to total MAPKs for each sample. The fold change of phospho-MAPKs levels in vehicle group was set at 1 for quantifications. Data were shown as mean ± SEM. *P < 0.05, **P < 0.01, ***P < 0.001 *vs*. vehicle.

In order to explore the mechanisms responsible for the therapeutic effect of YM_11_ on pain, MAPKs pathways, which play an important role in induced nociceptive pain and pathological pain^11, 12^ were investigated. The results showed that basal levels of p-p38, p-ERK and p-JNK were detected in vehicle, aspirin and YM_11_ groups, respectively. Among MAPKs pathways, the expressions of p-ERK were markedly decreased in all dose groups, the level of p-JNK was significant decreased only at high dose, and that of p-p38 were significantly decreased at both middle and high doses (Fig. 12). These results suggested that YM_11_ could decrease the level of MAPKs channel protein in a dose-dependent manner.

## Sodium currents recording

### Na_v_1.8 sodium current peak value and the change of the I-V curve

**Figure 13:**
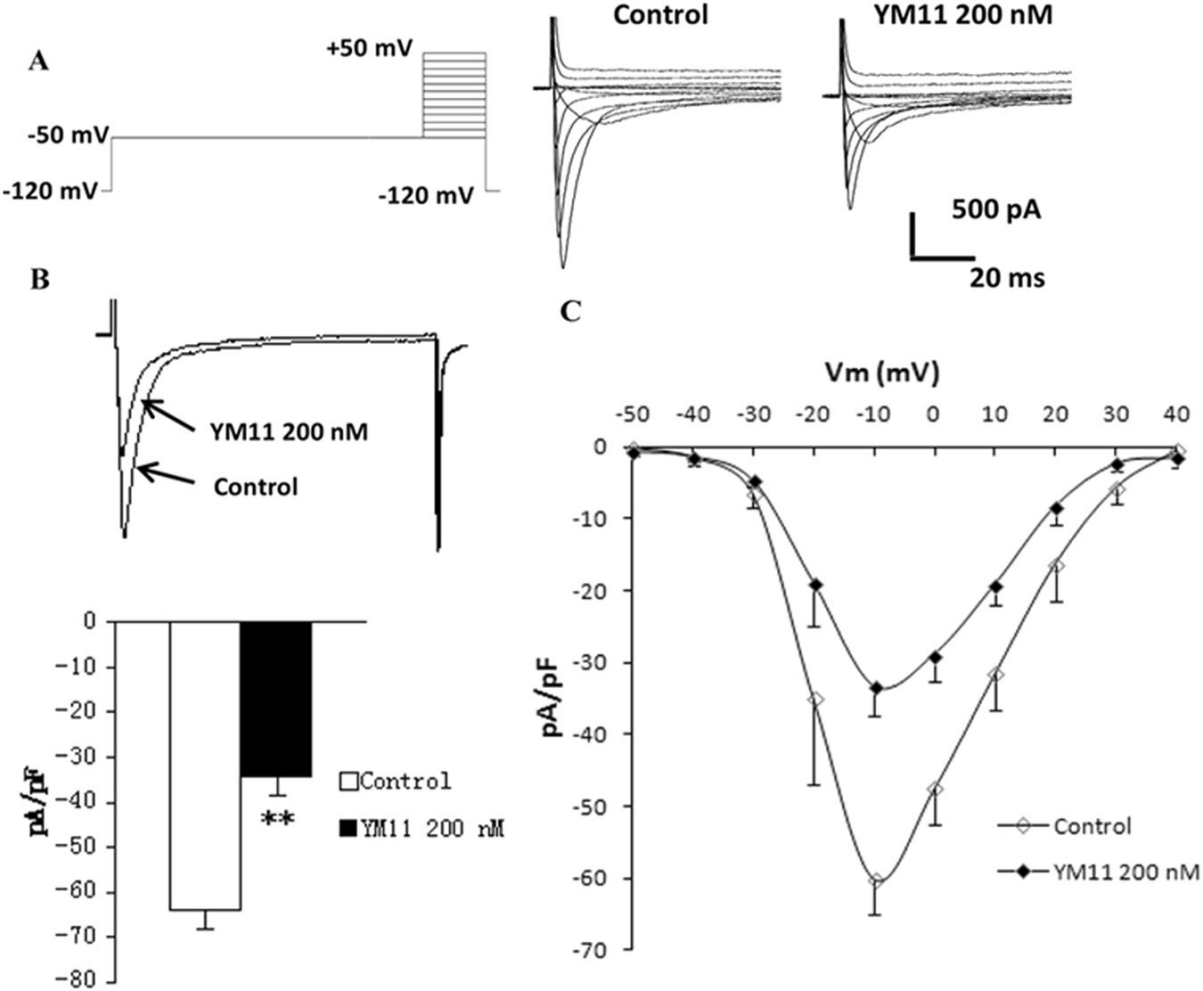
Changes in Na_v_1.8 sodium current following YM_11_ treatment. The Na_v_1.8 current was evoked by a depolarizing voltage steps from −50 to +50 mV in 10 mV increments from a holding potential of −120 mV (**A**). After YM_11_ treatment, the peak sodium current density mediated by Na_v_1.8 was significantly (**P < 0.01) decreased by 46.01% (**B**), but no change in *I-V* relationship (**C**).

Na_v_1.8 currents were recorded in small (<25 µm) neurons. The Na_v_1.8 current was evoked by a depolarizing voltage steps from −50 to +50 mV in 10 mV increments from a holding potential of −120 mV (Fig. 13A). After YM_11_ treatment, the peak sodium current density mediated by Na_v_1.8 was significantly decreased by 46.01% (*P* < 0.01) (Fig. 13B), but no change in *I-V* relationship (Fig. 13C).

### YM_11_ affect Na_v_1.8 sodium channel steady-state activation curve

**Figure 14:**
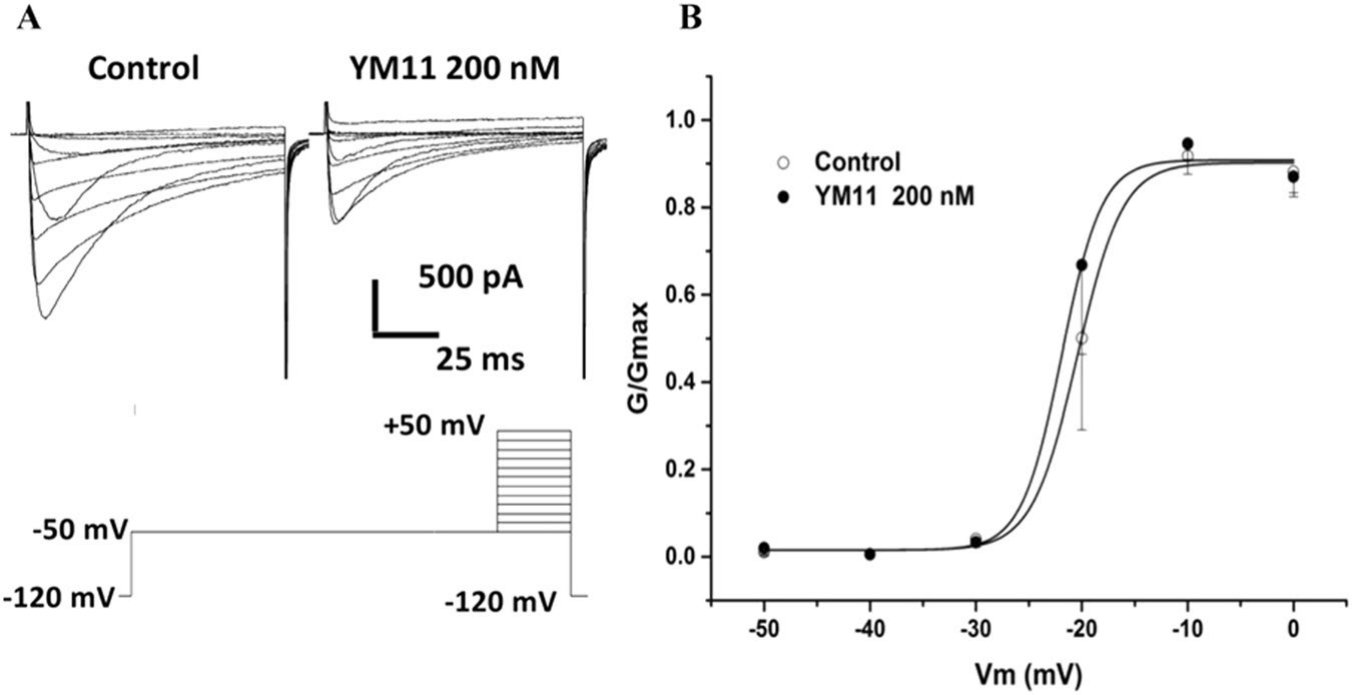
Changes of Na_v_1.8 steady-state activation curves among control. The values of the parameters of *V*_*1*/*2*_ and *K* were −20.42 ± 0.36 mV and 2.31 ± 1.26 (n = 5) under control condition, −21.90 ± 1.51 mV and 1.90 ± 1.43 (n = 5) in rat L4–6 DRG neurons.

The changes of Na_v_1.8 steady-state activation curves (Fig. 14) illustrated that the values of the parameters of *V*_*1*/*2*_ and *K* were −20.42 ± 0.36 mV, 2.31 ± 1.26 (n = 5) under control condition, and −21.90 ± 1.51 mV, 1.90 ± 1.43 (n = 5) in rat L4–6 DRG neurons after YM_11_ treatment.

## Discussion

It is known to all that pain experience incorporates both sensory and affective dimensions. The former describes the physical level and the latter comes from the emotional level. It is an extremely painful experience, and patients always suffer from fear, anxiety, anger, depression and even a suicidal tendency. Clinical observations indicate that the patients usually suffer from both emotional disturbance and pain sensation^13^. So far, pain has received more attention. The pharmacological treatment of patients with chronic pain is a current challenge, since existing drugs have little efficacy and present serious side effects as mentioned above.

The application of medicines derived from natural products is a field that has been drawing an increased interest in recent times. Up to now, plant-based agents have already played an important role in the treatment of various diseases and syndromes. China is famous for the long history application of plant medicines. Although some plant-based agents, such as Ginkgo biloba extract and total saponins of Ginseng have been widely accepted, there are still many folk medicines, especially those in some minority areas, are unknown to the world. So, it’s very necessary and urgent to carry out the systematic studies on these medical plants in order to find more safe plant-based agents to benefit the health of human beings. Therefore, as one of characteristic folk medicine in Dong Nationality of China, *S. brachyanthera* was selected as the current objective based on our previous investigations on Chinese ethnopharmacologial medicines in the central and south-west areas of China. A series of animal experiments were adopted to prove its analgesic activity and sustain its traditional usages.

In addition, just as discussed above, nor-oleanane triterpenoids are the main constituents of *S. brachyanthera*. In the current phytochemical study, all 11 isolated compounds from EESB were nor-oleanane triterpenoids, among which the content of YM_11_ was the highest that reached to gram grad. Therefore, in order to ensure the accuracy and reproducibility, YM_11_, the characteristic ingredient of the plant material, was selected for further investigations to evaluate its bioactivities with the aim of revealing the possible mechanisms.

Writhing response induced by acetic acid and hot-plate latent pain response in mice are two common and important models for screening analgesic. These two models could cause pain by liberating PGs and many others that excite pain nerve endings. In these two models, aspirin and morphine were used as positive drugs to study the peripheral and central analgesic activities, respectively. The results showed that EESB could provide responses to two different grades of noxious stimuli including chemically induced and thermal stimulus tissue damages. Considering the higher anti-inflammation effect of EESB than that of aspirin in anti-inflammatory test at doses of 400 mg/kg, it could be preliminary concluded that the possible mechanism of analgesic capacity of EESB might probably lie in the blockade the release of PGs and other cytokines that excite pain nerve endings, which is much alike aspirin.

Similarly, YM_11_ also significantly reduce the number of the body torsion in writhing test, and the inhibitory rate at dose of 20 mg/kg was higher than that of aspirin (71.87%). Therefore, YM_11_ had a non-steroidal anti-inflammatory drug like peripheral analgesic activity which was also confirmed by Randall-Selitto^14^. As we known that hot plate test is to evaluate the pain threshold of mice tolerance heat^15^ and is thought to measure the complex response to a non-inflammatory^16^. However, the results suggested that YM_11_ was not a centrally acting analgesic because no significant results were obtained. But EESB exhibited almost similar analgesic effect to morphine, and the inhibition rate (38.6%) was even stronger than that of morphine (35.7%) after 2 h, indicating its long persistent period of antinociceptive effect. This result demonstrated that, although single component may be not active, the co-existence of multi-components in the plant could strengthen the biological effects of the whole, and fully illustrated the multi-targets property and synergistic effect of traditional Chinese medicine.

Ear edema, one of the classic inflammatory models was induced by xylene, may involve inflammatory mediators, which can promote vasodilatation and increase vascular permeability^17^. In this study, the experimental results revealed that both EESB and YM_11_ could significantly inhibit the ear edema compared with control group with a dose-depended decrease effects. The maximum inhibition rate of YM_11_ was 21.4% at the dose of 80 mg/kg. Therefore, the current data confirmed the anti-inflammation effects of the extracts of *S. brachyanthera* and its main constituent, which might reduce the level of inflammatory mediators during the acute inflammation and pain response.

Formalin test is an effective model in pain and analgesia research^18^, which can produce distinct biphasic nociceptive responses^16^. The short-lived first response is thought to be produced by direct activation of nociceptive neurons by formalin and the second phase is thought to be a part of an inflammatory response to tissue injury^19, 20^. The first 5 min after formalin injection was known as the first phase, followed by a quiescent period of approximately 10 min, and then the second phase occurred from 15 to 60 min after this period. Generally speaking, centrally acting drugs inhibit both two phases equally^21^, while peripherally acting drugs inhibit the second phase^22^. In this test, although YM_11_ had the inhibitory effects in both two phases and the inhibitory effect at the second phase was more significant than that at the first phase. That’s to say, the present results revealed remarkable antinocifensive effect of YM_11_ on phase II, which was similar to that of aspirin. Therefore, combining with our previous experimental results in writhing response and hot plate test, it was suggested that the analgesic mechanism of YM_11_ tends to be peripheral analgesia.

While previous studies have provided compelling evidence that pathological pain pathogenesis is not only simply referred to the changes of the activity of neuronal systems, but also involves MAPK path, inflammatory immune and so on^23^. The mitogen-activated protein kinase (MAPK) is a family of serine/threonine protein kinases that play critical roles in regulating neural plasticity and inflammatory that could transduce extracellular stimuli into intracellular posttranslational and transcriptional responses^24–26^. It includes three major members: extracellular signal-regulated kinases (ERK), p38, and c-Jun N-terminal kinase (JNK), which represent three separate signaling pathways^12^. Recent studies indicated that all three MAPK pathways contribute to pain sensitization, and phosphorylation of MAPKs under different persistent pain conditions could lead to the induction and maintenance of pain hypersensitivity^12, 27^. So, the inhibition of MAPKs could reduce the inflammatory reaction and pain response^28–30^. JNK and p38 are the main mediators of cellular stresses, inflammation and physiological functions, and a large number of evidence indicated that they also contribute to pain hypersensitivity^11, 31^. More evidences demonstrated that ERK is involved in neuronal plasticity, such as pain hypersensitivity. Besides, ERK activation (phosphorylation) in neurons depends on nociceptive activity^32^. In our present study, it could be found that YM_11_ could significantly inhibit the expressions of p-JNK, p-ERK, and p-p38 at all doses in which the phosphorylations of three proteins declined significantly. The results also showed that the productions of IL-1β, IL-6 and TNF-α were also inhibited significantly by YM_11_ in a dose-depended manner, indicating its effects on pro-inflammatory cytokines. More importantly, all of these results of YM_11_ on proteins and pro-inflammatory cytokines were consistent with the experimental results of animal behaviors, which proved the possible analgesic mechanism of YM_11_ was to decrease the production and release of pro-inflammatory cytokines by inhibiting the MAPK signal transduction pathway. Because once MAPKs pathway was activated, pro-inflammatory cytokines such as TNF-α and IL-1β will be released from astrocytes that result in pathological pain^33^. Besides TNF-α, IL-1β and IL-6 can also been detected at the injury site, and shows temporal up-regulation in neuropathic pain^34–36^. Some reports also confirmed the effects of these important pro-inflammatory cytokines during the development of pain^37–39^. Therefore, the present studies confirmed this viewpoint in a further step that the inflammatory mediators would participate in the expression of MAPKs pathways and the sodium channels.

Moreover, the use of sodium channel blockers to treat both neuropathic and inflammatory pain in a clinical setting is well known to result in analgesia^40–42^. In the sensory primary afferent neurons, the voltage-gated sodium channels (VGSCs) are considered to play a critical role in the pathogenesis of chronic pain conditions^43, 44^. According to their sensitivities to tetrodotoxin (TTX), a kind of specific channel blocker, and the dynamics characteristics, VGSC are divided into TTX-sensitive (TTX-S) and TTX-resistant (TTX-R) sodium currents^45^. Among them, Na_v_1.8 and Na_v_1.9 are the two major VGSCs specifically locating on small (with small and medium diameter) sensory neurons that are closely associated with the nociceptor, in which Na_v_1.8 is mainly involved in formation of upstroke velocity of the action potential, while Na_v_1.9 will promote the formation of resting potential. The evidence suggested that Na_v_1.8 mainly contributes to the development of peripheral sensitization and the enhanced of analgesic effect in DRG neurons. In addition, the studies also indicated that the regulation of Na_v_1.8 can also promote the maintenance of inflammatory hyperalgesia. Therefore, the effects of YM_11_ on Na_v_1.8 were investigated, and the results demonstrated that the significantly increasing of TTX-R sodium currents of small-sized DRG neurons isolated from mice in formalin test (Na_v_1.8 in L_4–6_ DRGs) could be efficaciously ameliorated after the administration of YM_11_. Therefore, combined with the results of MAPKs, it could be believed that the analgesic effect of YM_11_ mainly comes from the suppression of the expressions of Na_v_1.8 in L_4–6_ DRGs due to the inhibitions of p38, JNK and ERK. This result was identical with the previously reported^46, 47^, namely, the activation of MAPKs pathway could affect the downstream of a variety of regulatory proteins, in turn, control the downstream of sodium channel, which are responsible for the generation and propagation of action potentials in excitable cells in response to membrane depolarization.

In conclusion, our research work provided the first systematical report of pharmacological activities of the EtOH extracts of *S. brachyanthera* (EESB) and YM_11_, which offered new research direction for analgesic drugs. It must be pointed out that in our research, the anti-inflammatory and analgesic activities of EESB on pathological pain, especially on peripheral (inflammatory) analgesia in experimental animal models is basically same to YM_11_, whose amount was the highest in the plant. Moreover, different kinds of substituent groups or functional groups in isolated compounds might be worth of further investigation and elucidation for their biologically activities, which are in progress in our research team. Therefore, it could be desirable that some analgesic leading compounds with fewer negative side effects would be obtained from *S. brachyanthera*. The results from current studies on YM_11_ comprehensively indicated that the mechanisms were close related to the direct reduction of the sodium channel current density, the inhibitions of expression of sodium channel protein regulated by MAPKs, and productions of pro-inflammatory cytokines including TNF-α, IL-1β and IL-6.

## Materials and Methods

### Plants

*S. brachyanthera* was collected in Hunan Province by Shumo Mei, Huaihua Medical College on October 2009, and was identified by Pro. Jincai Lu, School of Traditional Chinese Material Medica, Shenyang Pharmaceutical University. A voucher specimen (NO.HLG-0910) is deposited in School of Traditional Chinese Material Medica, Shenyang pharmaceutical University.

### Preparation of the extracts and the isolation of the compounds

The air-dried stems of *S. brachyanthera* (7.0 kg) were chopped into small pieces and then extracted three times with 70% EtOH under reflux for 2 h. After evaporation of the combined EtOH extracts *in vacuo*, the resultant aqueous residues were heated into dryness to get the extracts (EESB, 1652 g, 23.6%). After successively extracted by EtOAc and n-BuOH, the n-BuOH soluble fractions (175 g) were collected and then submitted to SiO_2_ column chromatography (CC) (φ1000 mm × 100 mm) and open ODS CC(φ500 mm × 60 mm) successively, and then separated by reverse-HPLC with 45% MeOH-H_2_O as mobile phase to get compound **1** (25.2 mg), compound **2** (18.6 mg), compound **3** (221.2 mg), compound **4** (19.2 mg), compound **5** (27.3 mg), compound **6** (28.6 mg), compound **7** (27.9 mg), compound **8** (179.1 mg), compound **9** (1100.1 mg), compound **10** (5,806.2 mg), and compound **11** (69.1 mg), respectively. The extract and selected compound were dissolved in 0.5% CMC, respectively, to make up different concentrations prior to administration to experimental animals.

### Animals

Healthy Kunming mice (18–22 g) and SD rats (150–160 g), obtained from the Experimental Animal Center of Shenyang Pharmaceutical University, Shenyang, China, were kept at 25 ± 1 °C under a 12 h light/dark cycle and were fed standard laboratory diet and water ad libitum. All animals’ treatment protocols were seriously in accordance with the international ethical guidelines and the National Institute of Health Guide concerning the Care and Use of Laboratory. Animals and all experiments were performed on the basis of the approved protocols of Animal Ethics Committee, Shenyang Pharmaceutical University, China (SCXK (Liao) 2010–0001).

### Instruments and reagents

Mettler electronic balance (Mettler-drag bullish Instrument Co., Ltd.), Hot plate (Shanghai Precision Instrument Co., Ltd.), syringe (Shanghai Kaile Infusion Factory), Ophthalmic scissors (Shanghai Medical Devices Co., Ltd.), High-speed centrifuge thermostat (US beckman company J2-HS), Glue means (US Bio-Rad), Electrophoresis tank (vertical) (US Bio-Rad 552BR061900), Transfer membrane device (wet) (US Bio-Rad 153BR51853), Electrophoresis (Shanghai-day energy company 041BR82104), Horizontal shaker (Beijing sixty-one Instrument WD-9405B), Organization sonicator (Ningbo Biotechnology Co., Ltd.), Multifunctional microplate reader (BWTeK company 70905568), Water bath (Changzhou Guohua Electric Co., Ltd.), Magnetic stirrer (Jiangsu Province Jintan Ronghua Instrument Co., Ltd.), pH meter (Sartorius), Inverted microscope (Nikon), Patch clamp amplifier(Axon), Number-analog converter system (Axon), Three-dimensional hydraulic micromanipulator (Japanese NARISHIGE).

Xylene, acetic acid and sodium carboxymethyl cellulose were purchased from Merck Co., Germany. Aspirin used as reference were obtained from Tolid Daru Co., Tehran, Iran. Anti-Nav1.8, Glycine, 2-Mercaptoethanol, APS, TEMED, PMSF were purchased from Amresco. IL-1β Kits, IL-6 Kits and TNF-α Kits were purchased from Sigma-Aldrich (Sigma-Aldrich China, Shanghai, China). All chemicals and solvents used in this study were of analytical grade.

NMR spectra were acquired using Bruker ARX-400 and ARX-600. Chemical shifts (*δ* ppm) are relative to TMS as an internal standard. Preparative HPLC was employed on an YMC-ODS column (YMC-Pack ODS-A, 250 × 20 mm) with a refractive index detector (SPD-6A). Column chromatographic (CC) purifications were carried out on silica gel (200–300 mesh) (Qingdao Haiyang Chemical Group Corporation, China) and ODS (YMC-Pack-ODS, 10–30 mm) respectively.

### Acetic acid-induced writhing response in mice

The method of Koster *et al*.^48^ was used for this test. The tested male mice were respectively given EESB, YM_11_, aspirin, and vehicle (i.g.) at 0.1 mL/10 g body weight twenty minutes prior to an intraperitoneal injection of 0.7% acetic acid at 0.1 mL/10 g body weight. The writhings induced by the acid, consisting of abdominal constrictions and hind limbs stretchings, were counted for 30 min. The percentage of inhibition was calculated using the following formula: %Inhibition = (Writhing Number_vehicle_ − Writhing Number_drug_)/Writhing Number_vehicle_ × 100%.

### Hot-plate latent pain response test in mice

The hot plate test was performed as described in literature^49^. The temperature of the hot-plate was set at 55 ± 1 °C. Each female mouse was placed on hot-plate to observe its pain responses (hind-paw-licking or jumping) acted as its own control. The latent time before the occurrence of the pain response was recorded as an analgesic parameter. The mice whose latent response times were shorter than 5 s or longer than 30 s were excluded from the test. The vehicle, positive control, EESB, and YM_11_ were then intragastrically administrated to mice and the hot-plate latent response time of each animal in one minute was recorded at 15 min, 30 min, and 60 min, respectively. The percentage analgesic activity was calculated as follows: %Baseline latency increase = (Baseline Latency_drug_ − Baseline Latency)/Baseline Latency × 100%.

### Xylene-induced ear edema in mice

According to the method previously described^50^, EESB, YM_11_ and aspirin were given orally to the male mice. 40 min later, each animal received 20 μL of xylene on the left ear was considered as control without treatment. Mice were sacrificed by cervical dislocation 1 h after xylene application. Ear biopsies of 7.0 mm in diameter were punched out and weighed. The extent of edema was evaluated by the weight difference between the right and the left ear disks of the same animal. The inhibition of edema in percentage was calculated as follows: %Inhibition = (ear edema_control_ − ear edema_drug_)/ear edema_control_ × 100%.

### Formalin test

The formalin test was carried out as reported^51^. Each test group of male mice were intragastric administrated YM_11_, EESB, vehicle, control and aspirin at 0.1 mL/10 g body weight forty min prior to receive 20 μL of 2.5% formalin solution, injected in subcutaneously in the ventral right hind paw. After the formalin injection, animals were put back into the glass cone and were observed for 60 min. A mirror was placed behind the glass cone to allow an unobstructed view of the formalin injected paw. The time (five min) that animals spent licking, shaking and retracting the injected paw was timed with a chronometer and was considered to be indicative of ongoing nociception.

### Biochemical parameters

Ear tissue of mice (from xylene-induced ear edema in mice model) was added into the PBS buffer solution under the ice bath to prepare by sonication. After centrifugation, the supernatants were used to measure the concentrations of proinflammatory cytokines using corresponding ELISA kits.

The expression levels of IL-1β, IL-6 and TNF-α in supernatants were analyzed using an ELISA immunoassay according to the manufacturer’s instructions. Briefly, proinflammatory cytokines level was measured by pipetting 50 µL of standard and put in 10 µL of sample and 40 µL of sample diluents into the wells of a microtiter plate coated with an antibody specific to right antibodies, subsequently covered with an adhesive strip and incubated for 1 h at 37 °C. After five times washing with Wash Solution, 50 µL of chromogen solution A and 50 µL of chromogen solution B were mixed and incubated for 10 min at 37 °C continually, protected from light. The color in the wells was changed from blue to yellow after 50 µL of stop solution was added. In the end, the optical density was calculated with an automated coulter microplater reader at 450 nm. The corresponding sample concentration was calculated according to the OD value.

### Extraction of protein and Western Blot Analysis

Protein immunodetection was carried out as previously described^52^. Bilateral L4-L6 DRGs from formalin experiment of mice were dissected and total proteins were extracted via homogenization in ice cold lysate buffer and then centrifuged at 4 °C for 15 min at 12000 rpm. Subsequently, the supernatant (total protein) was collected, and the protein levels were quantified using a BCA assay.

Soluble antigens (the target protein) were loaded per lane, which were electrophoretically separated using 12% denaturing poly-acrylamide gel electrophoresis (SDS-PAGE). After the separation, the proteins were transferred from the gel to nitrocellulose membrane. The membranes were blocked in 5% nonfat milk in TBST buffer (Tris Buffer Saline containing 0.1% Tween-20) for 1 h at room temperature. Then, took out membranes and added first antibody overnight at 4 °C. After extensive washing with TBST buffer, second antibody was then added. after 30 min gently rocking, the second antibody solution were pour off and the target proteins were detected by enhanced chemiluminescence detection of the proteins using an ECL kit. The band densities were quantified with a computer-assisted imaging analysis system (Syngene) with β-actin as an internal control.

## Sodium currents recording

### Solutions preparation

D-Hank’s: 0.2 g KCl, 4.0 g NaCl, 0.067 g Na_2_HPO_4_·12H_2_O, 0.03 g KH_2_PO_4_, and 0.175 g NaHCO_3_ were dissolved into 500 mL deionized water, adjusted pH value to 7.2, and sterilized under 121 °C for 40 min and then saved at 4 °C.

Polylysine: 10 mg Poly lysine were dissolved in 100 mL deionized water, then filtratied through 0.22 µm microporous membrane and remained at 4 °C.

Dorsal root ganglion (DRG) neurons were isolated firstly from anesthetized rats and then cut into pieces. After digested with 2.0 mL Digestive juices for 15 min at 37 °C, the DRG fragments were rinsed three times with culture medium of fetal bovine serum to stop enzymatic digestion. Then subsequently, the medium containing DRG neurons was vaccinated in the petri dish. At last, the dish put in incubator for 1 h.

### Sodium currents recording

The current recording was proceed at room temperature. Small DRG neurons (diameter 15~25 μm) with smooth and intact cell membranes, oval or round cell body, good refraction, and strong sense of three-dimensional were chosen. The pipette solution contained (in mM): 100 CsCl, 30 CsF, 8 NaCl, 2.4 CaCl_2_, 1 MgCl_2_, 5 EGTA, 4 Na_2_ATP, 10 HEPES, 0.4 GTP (pH 7.3). The bathing solution contained (in mM): 100 choline chloride, 40 NaCl, 3 KCl, 2.5 CaCl_2_, 1 MgCl_2_, 10 HEPES, 10 glucose, 0.005 LaCl_3_ (pH 7.4), supplemented with 1 μM TTX when the currents of TTX-R channel were recorded. Osmotic pressure of solutions was about 295–305 mOsm. The current was recorded by amplifier (Axon Instruments) and the recording data was acquired and analyzed by Clampex 10.0 and Clamfit 10.0 software. The sampling rate was 10 kHz. The “Pipette offset” was used to return the liquid junction potential to zero. After the whole cell patch clamp was established, the DGR neurons were held at −50 mV and given step voltage stimulation (a group of square-wave voltage from −50 mV to +50 mV, in 10 mV increments, 100 ms wave width). YM11 was dissolved in the bath solution. Throughout the experiment, bath or YM11 solutions were delivered to the DRG neurons via a fast gravity-driven perfusion system.

### Statistical analysis

The voltage dependence of activation was determined using standard protocols. The conductance *G*(*V*) was calculated according to *G* = *I*/(*V* − *V*_*Na*_), where *V*_*Na*_ is the reversal potential, *V* is the test pulse potential and *I* is the current amplitude. Normalized peak conductance was fitted by the following Boltzmann equation: *G*/*G*_max_ = 1/{1 + exp [(*V*_1/2_ − *V*)/*k*]}, where *G*_max_ is the maximum conductance, *V*_1/2_ is the membrane potential of half-maximal activation and *k* is the slope factor.

The voltage-clamp data were digitized and analyzed using pClamp 10.0 software (Axon Instruments). Multiple comparisons were evaluated by one-way ANOVA followed by Tukey’s test, two-way ANOVA or repeated-measure two-way ANOVA followed by Bonferroni test. When only two groups were compared, an unpaired t-test was used. Data were expressed as mean ± standard error of mean (SEM). *P* < 0.05 indicated statistically significant differences.
